# TGFβ1- miR-140-5p axis mediated up-regulation of Flap Endonuclease 1 promotes epithelial-mesenchymal transition in hepatocellular carcinoma

**DOI:** 10.18632/aging.102140

**Published:** 2019-08-10

**Authors:** Chuanfei Li, Di Zhou, Hao Hong, Shuangyan Yang, Li Zhang, Shiying Li, Peng Hu, Hong Ren, Zhechuan Mei, Hui Tang

**Affiliations:** 1 Department of Gastroenterology, The Second Affiliated Hospital of Chongqing Medical University, Chongqing 400010, China; 2 Department of Radiology, The First Affiliated Hospital of Chongqing Medical University, Chongqing 4001016, China; 3 Department of Orthopaedics, The Second Affiliated Hospital of Chongqing Medical University, Chongqing 400016, China; 4 Department of Infectious Diseases, Institute for Viral Hepatitis, The Key Laboratory of Molecular Biology for Infectious Diseases, Chinese Ministry of Education, The Second Affiliated Hospital of Chongqing Medical University, Chongqing 400010, China

**Keywords:** Flap Endonuclease 1, miR-140-5p, EMT, hepatocellular carcinoma, TGF-β1

## Abstract

Flap Endonuclease 1 (FEN1) is a known oncogene in an array of cancers, but its role in hepatocellular carcinoma (HCC) remains obscure. In this study, we report that FEN1 expression was elevated in the Cancer Genome Atlas (TCGA) database which was verified in HCC tissue and hepatoma cell lines. Pearson correlation analysis indicated that FEN1 was involved in HCC metastasis. We demonstrated that FEN1 silencing inhibits HCC cell epithelial-mesenchymal transition (EMT), invasion and migration *in vitro* and significantly suppressed tumor growth and metastasis *in vivo*. Conversely, FEN1 overexpression in HCC cells enhanced these metastatic processes. We further confirmed that FEN1 was a direct target of miR-140-5p, which was down-regulated in HCC tissues, and negatively correlated with FEN1 expression. Moreover, low miR-140-5p levels and high FEN1 expression predicted a poor clinical outcome. The effects of FEN1 overexpression could be partially abolished by miR-140-5p. miR-140-5p down-regulation and FEN1 overexpression were observed in a TGFβ1 induced EMT model. TGFβ1 mediated EMT could be blocked by miR-140-5p overexpression or FEN1 silencing. Taken together, our findings suggest that FEN1 is regulated by the TGFβ1- miR-140-5p axis and promotes EMT in HCC.

## INTRODUCTION

Hepatocellular carcinoma (HCC) is a highly malignant cancer that ranks third amongst cancer-related mortalities [1]. Metastasis is a complex process that leads to reduced cell adhesion, tumor cell transformation, growth, angiogenesis, invasion, dissemination, cell survival in the circulation, and colonization at secondary organs or tissues [2, 3].

The mechanisms of HCC metastasis remain largely undefined. Epithelial-to-mesenchymal transition (EMT) is a critical biological process involved in wound healing, tissue regeneration, inflammation [4], cancer metastasis [5] and the formation of cancer stem cells [6]. The process of EMT involves a loss of epithelial cell polarity, which enables cells to switch from an epithelial phenotype to an invasive mesenchymal state [7, 8]. Transforming growth factor-β (TGF-β) regulates EMT through SMAD-dependent and independent signaling [9, 10]. These pathways activate EMT related transcription factors, including Snail, Twist and Zeb, to trigger EMT, subsequently promoting the invasion and metastasis of tumor cells [11].

Flap Endonuclease 1 (FEN1) gene is located on human chromosome 11q12 [12], and plays a role in DNA replication repair [13], DNA synthesis [14], non-homologous end joining and homologous recombination [15, 16]. FEN1 is essential for the maintenance of genome stability and is associated with the onset and progression of various cancers including lung [17], breast [18], gastric [19], prostate [20] and pancreatic cancer [21]. FEN1 expression correlates with cancer grade, aggressiveness and survival [22, 23]. In addition, the FEN1 polymorphisms –69 G > A and 4150 G > T correlate with the risk of HCC [24]. Missense mutations in exon 2 within the FEN1 nuclease core domain have been observed during HCC development in the Korean population [25]. However, the functional significance of FEN1 during HCC metastasis has not been elucidated.

The present study aimed to investigate the biological function and the underlying mechanism(s) of FEN1 in HCC metastasis. Herein, we demonstrated that FEN1 was upregulated in HCC and its overexpression predicted poor prognosis. FEN1 overexpression was further confirmed in hepatoma cell lines and we demonstrated its ability to promote hepatoma cell metastasis *in vitro* and *in vivo* by inducing EMT. In addition, we showed that FEN1 was a direct miR-140-5p target, and its expression negatively correlated with miR-140-5p in HCC tissue. The ability of FEN1 to induce EMT was partially blocked by miR-140-5p *in vitro* and *in vivo*. The EMT phenotype and invasion induced by TGF-β could be reversed by the inhibition of FEN1 or upregulation of miR-140-5p. Thus, we propose FEN1 as a diagnostic biomarker of HCC and a potential target for anti-HCC therapy.

## RESULTS

### FEN1 is upregulated in HCC and is associated with poor prognosis

**Figure 1:**
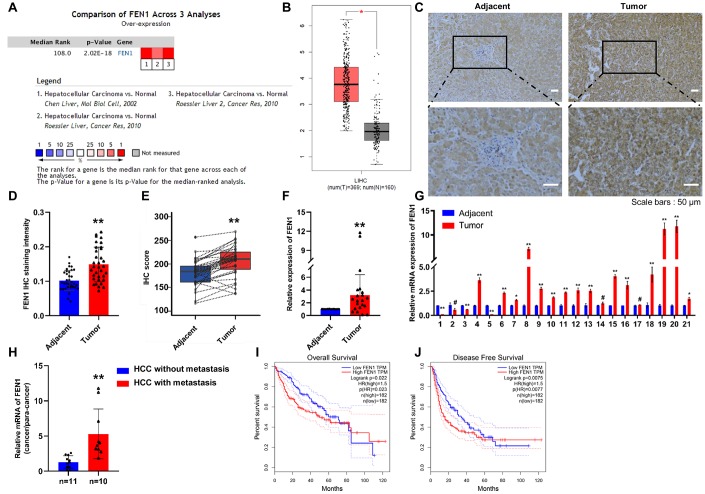
**FEN1 is up-regulated in HCC tissues and indicates poor prognosis.** (**A**) FEN1 mRNA expression in HCC among the three studies assessed by ONCOMINE analysis. (**B**) Elevated expression of FEN1 in HCC tissues compared to normal tissues in the GEPIA database. (**C**) FEN1 IHC in HCC and para-cancer tissues. (**D**) Quantification of FEN1 IHC staining; n = 34 per group, **P < 0.01. (**E**) FEN1 IHC score; n = 34 per group, **P < 0.01. (**F** and **G**) Quantitative RT-qPCR analysis of FEN1 mRNA expression; n = 21 per group, *P < 0.05, **P < 0.01, ^#^ represents no difference. (**H**) RT-qPCR analysis of FEN1 mRNA expression, n = 10 for HCC with metastasis and n = 11 for HCC without metastasis, **P < 0.01. High expression of FEN1 indicated poor overall survival (**I**) and disease free survival (**J**) in the GEPIA database.

A comparison of FEN1 mRNA levels in HCC from various studies in the ONCOMINE database revealed high FEN1 mRNA expression in HCC tissue (Figure 1A). Moreover, the expression of FEN1 in HCC was higher than that of non-tumor tissues according to the GEPIA database (Figure 1B). To verify FEN1 expression in HCC, we examined FEN1 expression in 34 cases of human HCC and matched adjacent tissues by IHC analysis. The results showed that FEN1 is primarily localized in the nucleus and that FEN1 expression significantly increased in HCC tissue compared to the adjacent para-tumor tissues (Figure 1C, 1D). The IHC score of FEN1 in HCC tissue was significantly higher than that in the tissue adjacent to the carcinoma (Figure 1E). RT-qPCR analysis in 21 pairs of matched HCC tissue and para-tumor tissue samples consistently showed a higher FEN1 mRNA expression in HCC tissues (Figure 1F). Specifically, 15/21 cases showed elevated FEN1 mRNA expression in HCC versus background non-cancerous liver tissue (Figure 1G). Association analysis showed that FEN1 expression significantly correlated with tumor size (P = 0.047) and metastasis status (P = 0.013), but did not correlate with patient gender, age, hepatitis B infection status, liver cirrhosis or tumor stage (Supplementary Table 4). We further found that the FEN1 mRNA levels in HCC with metastasis were notably higher than those in the HCC cases without metastasis (Figure 1H), indicating that FEN1 is involved in HCC metastasis. We confirmed these findings *in vitro*, showing that FEN1 mRNA and protein levels were significantly higher in the five human hepatoma cell lines (MHCC97-H, HepG2, SMMC-7721, HCCLM3 and Huh-7) compared to the normal HL7702 cells. Interestingly, the expression of FEN1 was higher in HCCLM3 cells which are more metastatic than MHCC97-H cells (Supplementary Figure 1A–1B).

We next investigated whether FEN1 overexpression was associated with clinical progression and outcomes in HCC using the GEPIA database. Kaplan–Meier analysis indicated that FEN1 expression was elevated in HCC tissues and was associated with reduced overall survival and disease free survival times (P = 0.023 and 0.0077) (Figure 1I, 1J). Taken together, these data reveal that FEN1 is a potential oncogene in HCC and its increased expression correlates with poor prognosis of HCC patients.

### FEN1 promotes EMT *in vitro*

To further characterize the function of FEN1 in HCC, SMMC-7721 cells were used to construct stable knock-down and overexpression cell lines. A total of three FEN1-shRNA lines were constructed and evaluated by RT-qPCR and WB analysis. FEN1 mRNA levels were found to be lower in cells expressing each FEN1-shRNA as compared to the control group (Supplementary Figure 1C). However, sh3-FEN1 produced the highest levels of FEN1 knockdown (Supplementary Figure 1D–1F). As expected, both FEN1 mRNA and protein expression were significantly up-regulated in FEN1 overexpressing SMMC-7721 cells compared to the control group (Supplementary Figure 1E–1F).

**Figure 2:**
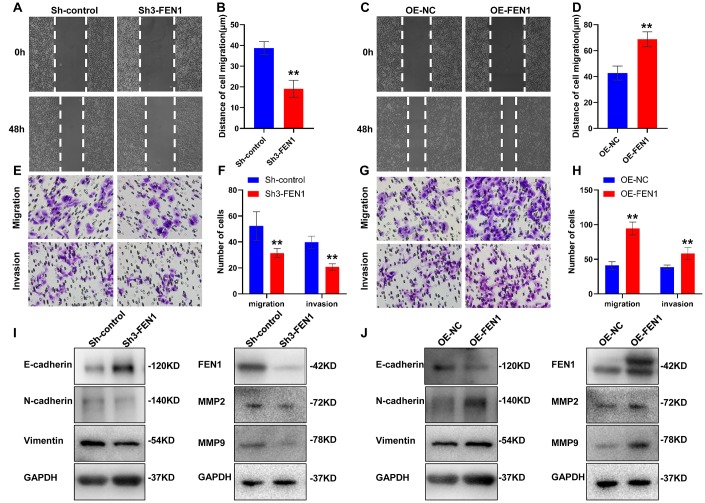
**FEN1 promotes HCC cell migration, invasion and EMT *in vitro*.** (**A** and **C**) Representative images of wound-healing assays and (**B** and **D**) quantification of wound closure. (**E** and **G**) Representative images of transwell chamber assays and (**F** and **H**) quantification of the number of cells which migrated or invaded through the basement membrane. Data represent mean ± SD of three independent experiments, **P < 0.01. (**I**) Western blot analysis of EMT markers, FEN1, MMP2 and MMP9 after silencing of FEN1. (**J**) Western blot analysis of EMT markers, FEN1, MMP2 and MMP9 after FEN1 overexpression.

Given the clinical significance of FEN1 in the HCC specimens, we assessed the effect of FEN1 on HCC cell migration, invasion and EMT using wound healing and transwell chamber assays. The wound healing capability of Sh3-FEN1 cells was significantly weaker at 48 h post-scratch compared to Sh-controls (Figure 2A, 2B). In contrast, cells overexpressing FEN1 (OE-FEN1) displayed significantly enhanced wound healing capability (Figure 2C, 2D). Similarly, Sh3-FEN1 cells showed significantly reduced migration and invasion capabilities as compared to Sh-controls in transwell assays (Figure 2E, 2F), whilst OE-FEN1 cells displayed significantly increased cell migration and invasion compared to OE-NC cells (Figure 2G, 2H).

We next assessed the effects of FEN1 on MMP2 and MMP9 expression. We found that FEN1 silencing reduced MMP2 and MMP9 expression (Figure 2I), while FEN1 overexpression enhanced MMP2 and MMP9 levels (Figure 2J). To directly assess the effects of FEN1 on EMT, we investigated the expression of EMT markers by WB analysis. Following FEN1 silencing, expression of the epithelial marker E-cadherin was significantly upregulated, whilst expression of the mesenchymal markers N-cadherin and vimentin were significantly downregulated compared to the negative controls. In contrast, a significant decrease in E-cadherin and a significant increase in N-cadherin and vimentin were observed following FEN1 overexpression (Figure 2I, 2J). Thus, these results indicate that FEN1 promotes hepatoma cell migration, invasion and EMT *in vitro*.

### FEN1 promotes tumor growth and lung metastasis *in vivo*

**Figure 3:**
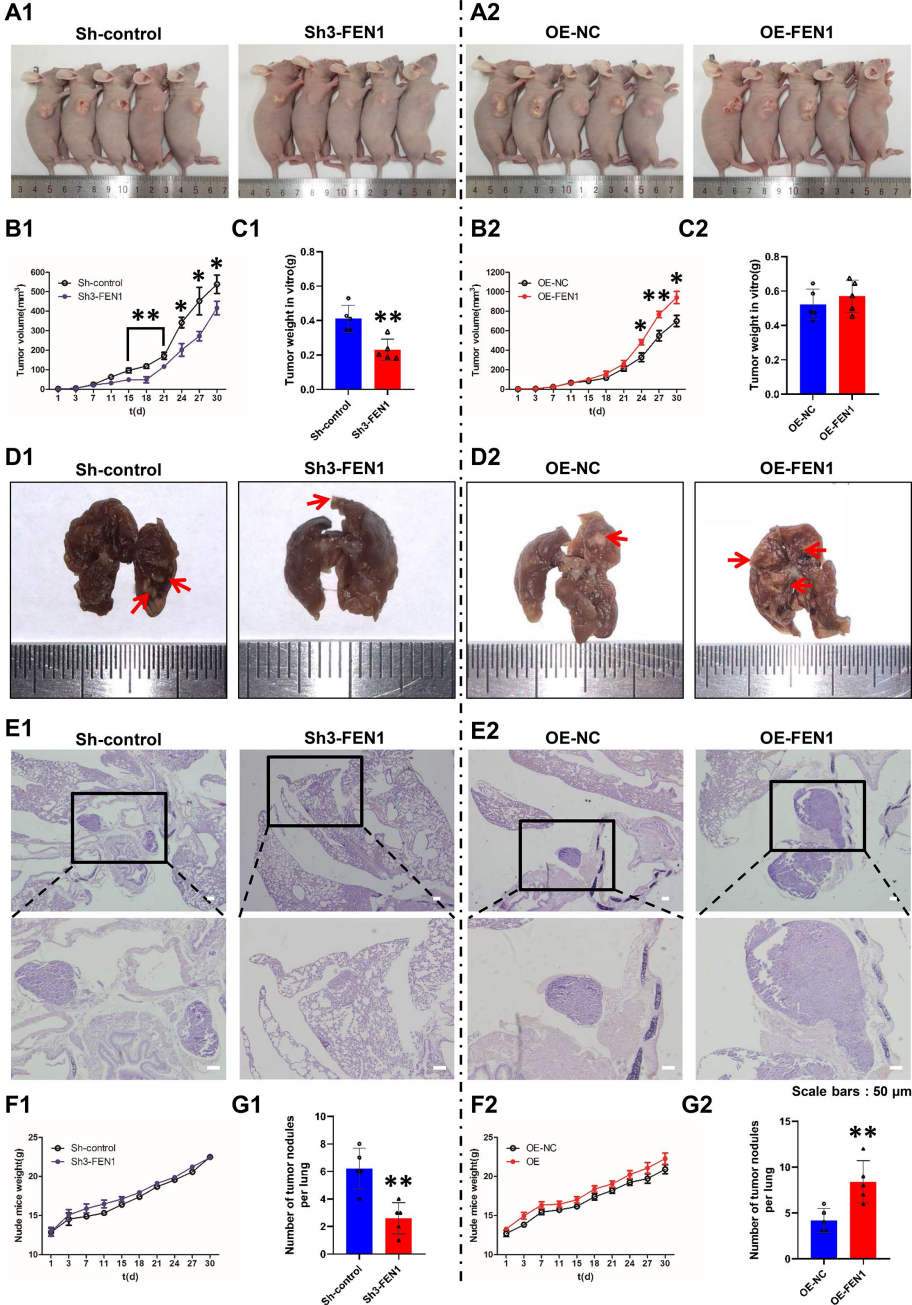
**FEN1 promotes tumor growth and lung metastasis *in vivo.*** (**A**) Images of nude mice bearing subcutaneous tumor xenografts derived either from Sh3-FEN1 (**A1**) or OE-FEN1 cells (**A2**). (**B1** and **B2**) Xenograft tumor growth curves; n = 5 per group, *P < 0.05 and **P<0.01. (**C1** and **C2**) Weights of tumors derived from euthanized nude mice at the endpoint; n = 5 per group, **P < 0.01. (**D1** and **D2**) Representative images of lungs with metastatic nodules shown on the surfaces (arrows indicate the nodules) 30 days after injection of cells stably silencing or overexpressing FEN1. (**E1** and **E2**) Representative H & E stained lung tissue sections showing metastatic nodules (arrows); original magnification, 100 x for the upper panels and 200 x for the lower panels. (**F1** and **F2**) Weight growth curves of nude mice, n = 5 per group. (**G1** and **G2**) Number of metastatic nodules on the lung surfaces; data represent mean ± SD, n = 5 per group, **P < 0.01.

To evaluate the effects of FEN1 on tumor growth *in vivo*, Sh-control, Sh3-FEN1, OE-NC and OE-FEN1 cells were injected into the armpits of nude mice (n=5 per group) and tumor sizes were measured every three days. Tumor growth curves were compiled and tumor weights were assessed on day 30 post-injection. We found that Sh3-FEN1 cell derived subcutaneous tumors grew significantly slower than Sh-control cell derived tumors (Figure 3A1, 3B1). The weight of the tumors obtained from the Sh3-FEN1 group was also significantly lower than those from the Sh-control group (Figure 3C1). Subcutaneous tumors derived from OE-FEN1 cells grew faster than the tumors derived from OE-NC cells (Figure 3A2, 3B2). No significant differences were observed in the weights of xenograft tumors between the OE-FEN1 and OE-NC group (Figure 3C2).

To extend our analysis to the effects of FEN1 on tumor metastasis *in vivo*, cells were injected into the tail veins of nude mice (n=5 per group). After four weeks, the Sh3-FEN1 group had significantly fewer metastatic nodules on the lung surface compared to the control group (Figure 3D1, 3G1). H & E stained lung tissue also showed fewer and smaller metastatic tumor nodules in the Sh3-FEN1 group compared to the control group (Figure 3E1). Moreover, significantly more metastatic nodules were evident in the OE-FEN1 group compared to the OE-NC group (Figure 3D2, 3G2). In addition, tumor nodules in the lung tissue of the OE-FEN1 group were larger and of a higher number than those of the OE-NC group (Figure 3E2). We observed no significant differences in the weights of nude mice in each group (Figure 3F1, 3F2). These results indicated that FEN1 alters tumor growth and metastasis *in vivo*.

**Figure 4:**
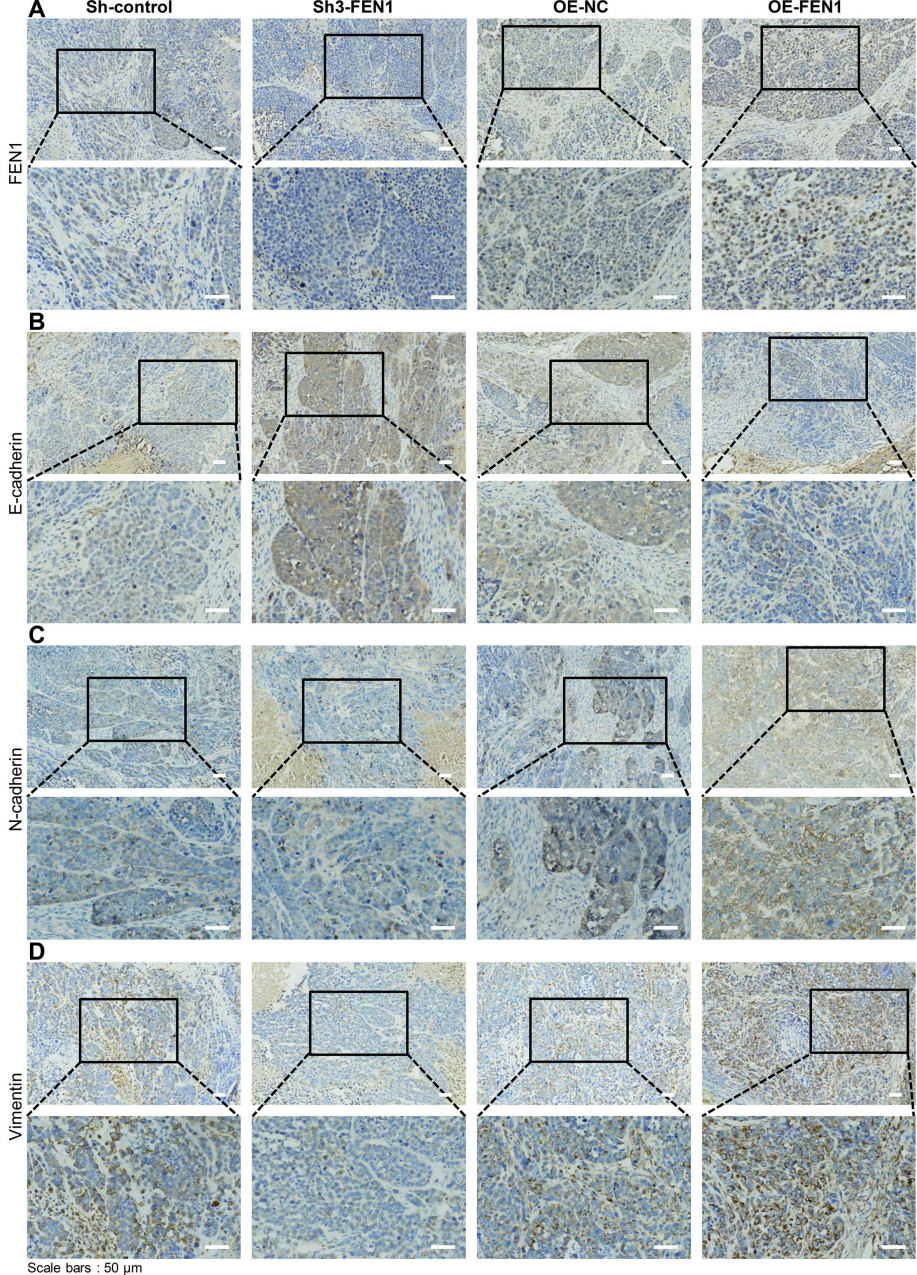
**FEN1 induces EMT *in vivo*.** (**A**) IHC staining of FEN1 in xenograft tumor tissue. (**B**) IHC staining of E-cadherin in xenograft tumor tissue. (**C**) IHC staining of N-cadherin in xenograft tumor tissue. (**D**) IHC staining of vimentin in xenograft tumor tissue; original magnification, 200 x for the upper panels and 400 x for the lower panels in each figure.

To confirm the effects of FEN1 on EMT *in vivo*, EMT markers were examined in the tumors of nude mice. IHC stained tumor tissue sections displayed lower FEN1 expression in the Sh3-FEN1 group and higher FEN1 expression in the OE-FEN1 group compared to the corresponding control groups (Figure 4A). We also observed dramatically higher levels of E-cadherin and decreased levels of N-cadherin and vimentin in the Sh3-FEN1 group compared to the control group. In addition, the expression of E-cadherin was lower, whilst the expression of N-cadherin and vimentin increased in the OE-FEN1 group compared to the OE-NC group (Figure 4B–4D). These results were consistent with the *in vitro* data and demonstrated that FEN1 promotes EMT *in vivo*.

### FEN1 is a direct target gene of miR-140-5p

**Figure 5:**
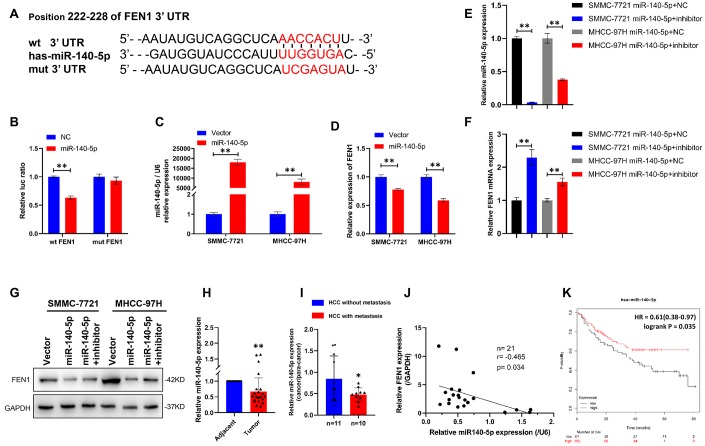
**FEN1 is a direct target of miR-140-5p in HCC cells.** (**A**) miR-140-5p and its putative binding sequence in the wild-type and mutant 3′-UTR of FEN1. (**B**) Overexpression of miR-140-5p significantly decreased the luciferase activity of constructs with wild type (WT) but not mutant (MUT) FEN1 3′-UTR in HEK-293 cells, **P < 0.01. (**C**) RT-qPCR showed that miR-140-5p mRNA was significantly increased in HCC cells transfected with lentiviral vector, **P < 0.01. (**D**) RT-qPCR showed that FEN1 mRNA was significantly decreased in HCC cells transfected with miR-140-5p vector, **P < 0.01. (**E**) Relative mRNA expression of miR-140-5p in HCC cells overexpressing miR-140-5p transfected with miR-140-5p inhibitors or NC, **P < 0.01. (**F**) Relative mRNA expression of FEN1 in HCC cells overexpressing miR-140-5p transfected with miR-140-5p inhibitors or NC, **P < 0.01. (**G**) Overexpression of miR-140-5p markedly suppressed FEN1 protein levels in HCC cells, and miR-140-5p inhibitors reversed the effect. (**H**) The expression of miR-140-5p in HCC tissues and the paired adjacent tissues was determined by RT-qPCR, **P < 0.01. (**I**) RT-qPCR analysis of miR-140-5p mRNA expression, n = 10 for HCC with metastasis and n = 11 for HCC without metastasis, *P < 0.05. (**J**) The correlation between miR-140-5p and FEN1 mRNA expression using Pearson’s correlation analysis. (**K**) Low expression of miR-140-5p indicated poor overall survival in the KM-plotter database.

To dissect the mechanism by which FEN1 promotes EMT, we performed bioinformatics analysis using the TargetScan dataset and found that FEN1 is a potential target of miR-140-5p (Figure 5A). To determine whether miR-140-5p bound to the predicted target site in the 3′-UTR of FEN1, we performed a miR-140-5p-based luciferase assay. As expected, miR-140-5p directly bound to the 3′-UTR of FEN1, and this binding remarkably reduced luciferase activity. In contrast, no significant reduction was observed when cells were transfected with mutant FEN1 3′-UTR constructs (Figure 5B). Moreover, miR-140-5p dramatically suppressed endogenous FEN1 levels in SMMC-7721 and MHCC97-H cells as measured by RT-qPCR and WB (Figure 5C, 5D, 5G). Consistent with these results, attenuating miR-140-5p expression rescued the suppression of FEN1 by miR-140-5p (Figure 5E–5G). These results indicated that FEN1 was a direct downstream target of miR-140-5p in HCC cells.

We further investigated the expression of miR-140-5p in HCC tissues by RT-qPCR analysis. As predicted, miR-140-5p expression was significantly lower in HCC tissue compared to para-cancerous tissue (Figure 5H). Additionally, the expression of miR-140-5p in metastatic HCC samples was markedly lower than that of HCC without metastasis (Figure 5I), indicating that miR-140-5p regulates HCC metastasis. Moreover, a negative correlation was observed between miR-140-5p and FEN1 (R = −0.465; P = 0.034; Figure 5J) in HCC samples. Kaplan–Meier analysis indicated a potential association between low miR-140-5p expression and poorer overall survival time in HCC patients (Figure 5K). Taken together, these findings suggest that miR-140-5p modulates HCC metastasis by targeting FEN1.

### Activation of miR-140-5p partially reverses the effect of FEN1 *in vitro* and *in vivo*

**Figure 6:**
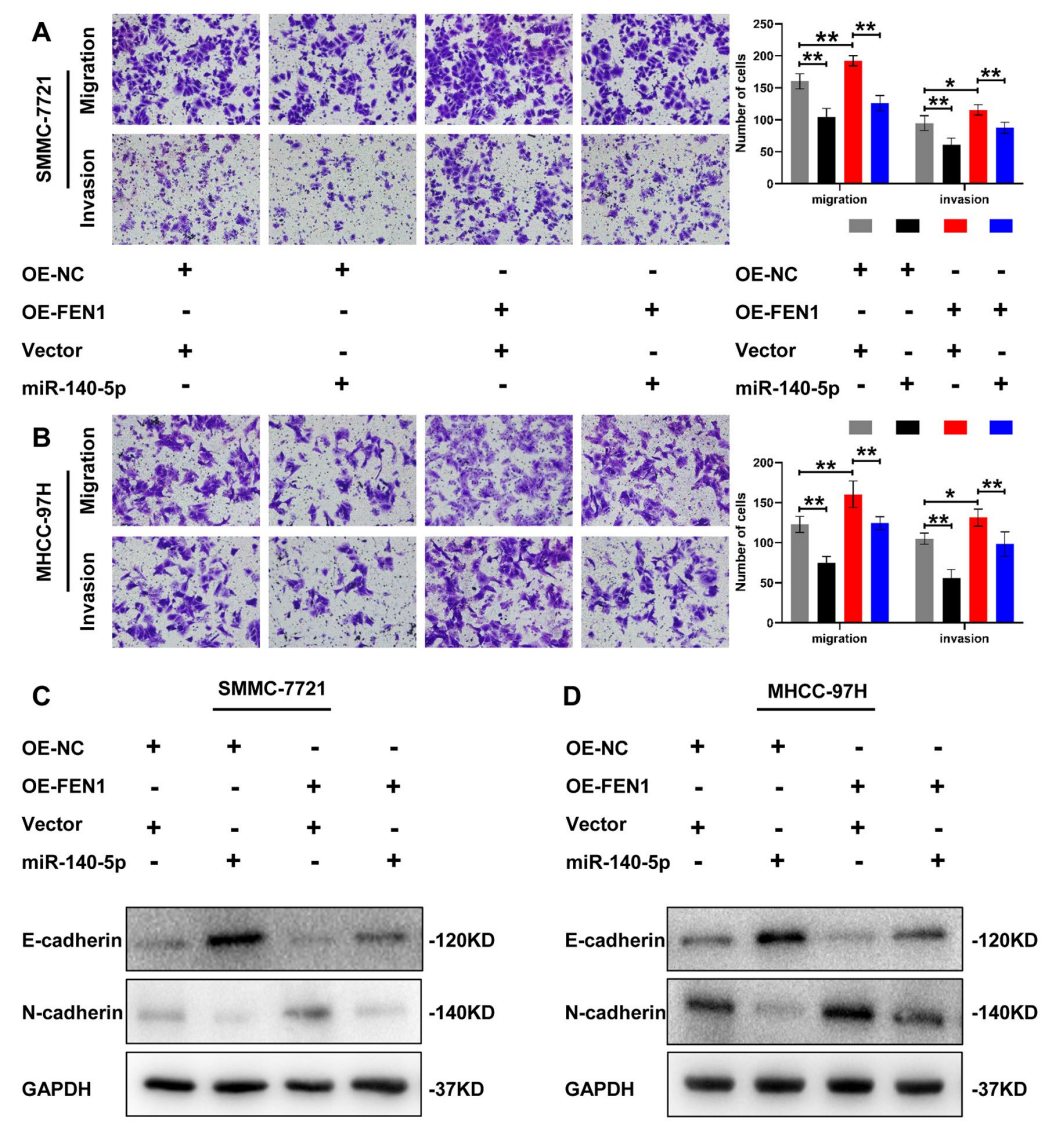
**miR-140-5p partially reverses the effect of FEN1 *in vitro*.** (**A** and **B**) Representative images of transwell chamber assays and quantification of the number of cells that successfully migrated or invaded through the basement membrane. Data represent mean ± SD of three independent experiments, *P < 0.05, **P < 0.01. (**C** and **D**) Western blot analysis of EMT markers in HCC cells overexpressing FEN1 which were transfected with miR-140-5p.

To determine whether miR-140-5p is involved in FEN1-promoted EMT, we first transfected MHCC- 97H and SMMC-7721 cells overexpressing FEN1 with miR-140-5p or NC. The effects of miR-140-5p on HCC cell migration, invasion and EMT were assessed by transwell chamber assays. The results showed that miR-140-5p significantly reduced the HCC cell migration and invasion induced by FEN1 overexpression (Figure 6A, 6B). Consistent with this result, FEN1-promoted EMT was also attenuated by miR-140-5p overexpression (Figure 6C, 6D). Together, these results indicate that miR-140-5p specifically blocks EMT via FEN1 *in vitro*.

**Figure 7:**
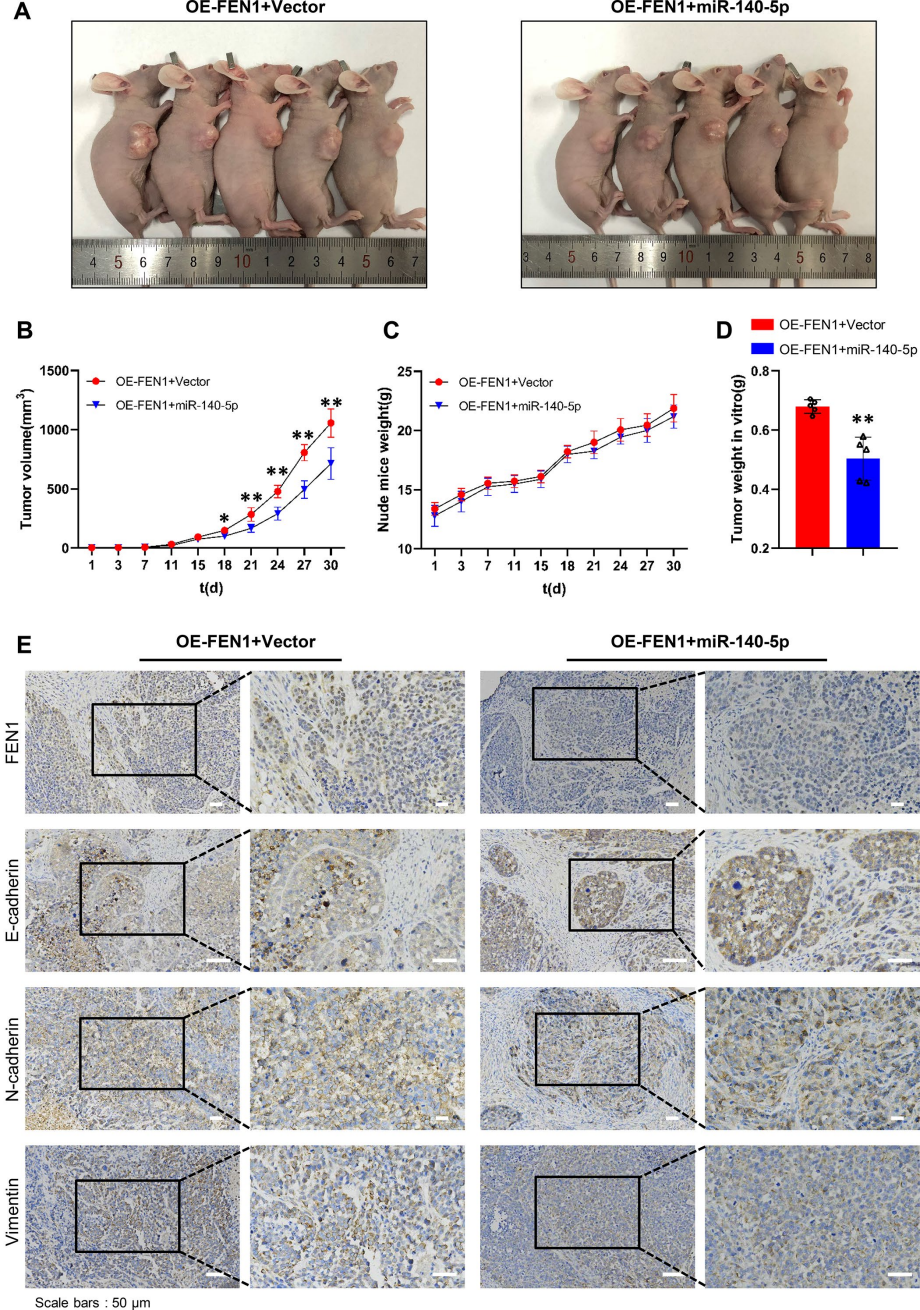
**miR-140-5p partially reverses the effect of FEN1 *in vivo*.** (**A**) Images of nude mice bearing subcutaneous tumor xenografts. (**B**) Xenograft tumor growth curves; n = 5 per group, *P < 0.05 and **P < 0.01. (**C**) Weight growth curves of nude mice, n = 5 per group. (**D**) Weights of tumors derived from euthanized nude mice at the endpoint; n = 5 per group, **P < 0.01. (**E**) IHC staining of FEN1 and EMT markers such as E-cadherin, N-cadherin and vimentin in xenograft tumor tissues; original magnification, 200 x for the upper panels and 400 x for the lower panels in each figure.

To evaluate the effects of miR-140-5p on tumor growth *in vivo*, a tumorigenic model was constructed in nude mice. The results showed that miR-140-5p significantly inhibited tumors growth in MHCC-97H cells stably overexpressing FEN1 (Figure 7A, 7B). In addition, final tumor weight was significantly reduced by miR-140-5p (Figure 7D). No significant differences were observed in the tumor weight growth curve (Figure 7C). Next, we examined the EMT markers in nude mice tumors by IHC analysis. The results demonstrated that the FEN1 expression was reduced by miR-140-5p. Additionally, FEN1 induced EMT was rescued by miR-140-5p (Figure 7E). These results were consistent with the *in vitro* data and demonstrated that miR-140-5p inhibits tumor growth and EMT by targeting FEN1 *in vivo*.

### TGF-β1 up-regulates FEN1 and down-regulates miR-140-5p

**Figure 8:**
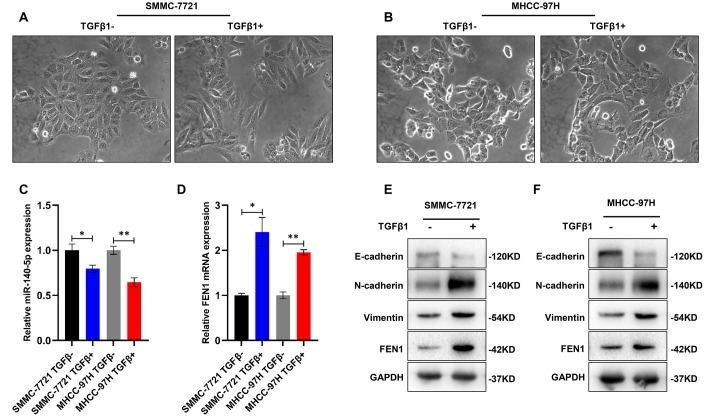
**TGF-β1 up-regulates FEN1 and down-regulates miR-140-5p.** (**A** and **B**) Representative images showing induction of morphological characteristics after 48 h of TGF-β1 (10 ng/mL) treatment. (**C** and **D**) RT-qPCR showed that miR-140-5p mRNA was significantly decreased in HCC cells treated with TGF-β1, and FEN1 was up-regulated in TGF-β1-treated HCC cells, *P < 0.05 and **P < 0.01. (**E** and **F**) Western blot analysis of EMT markers and FEN1 in TGF-β1-treated HCC cells.

Various cytokines and growth factors, including TGF-β are key agents for the initiation and maintenance of EMT [26]. Of the three identified TGF-β isoforms, TGF-β1 is the most significant inducer of EMT. EMT was therefore activated through the addition of recombinant human TGF-β1 to SMMC-7721 and MHCC-97H cells. The cells displayed obvious morphological changes including a spindle-like, fibroblastic phenotype after 48 h (Figure 8A–8B). WB analysis confirmed the EMT phenotype through the decreased levels of E-cadherin and increased levels of N-cadherin and vimentin (Figure 8E, 8F). Of note, FEN1 expression was significantly higher in TGF-β1 treated cells, whilst the miR-140-5p levels decreased (Figure 8C–8F), indicating a potential involvement of FEN1 and miR-140-5p in TGF-β1 signaling.

### Silencing FEN1 partially reverses TGF-β1- triggered EMT in HCC cells

**Figure 9:**
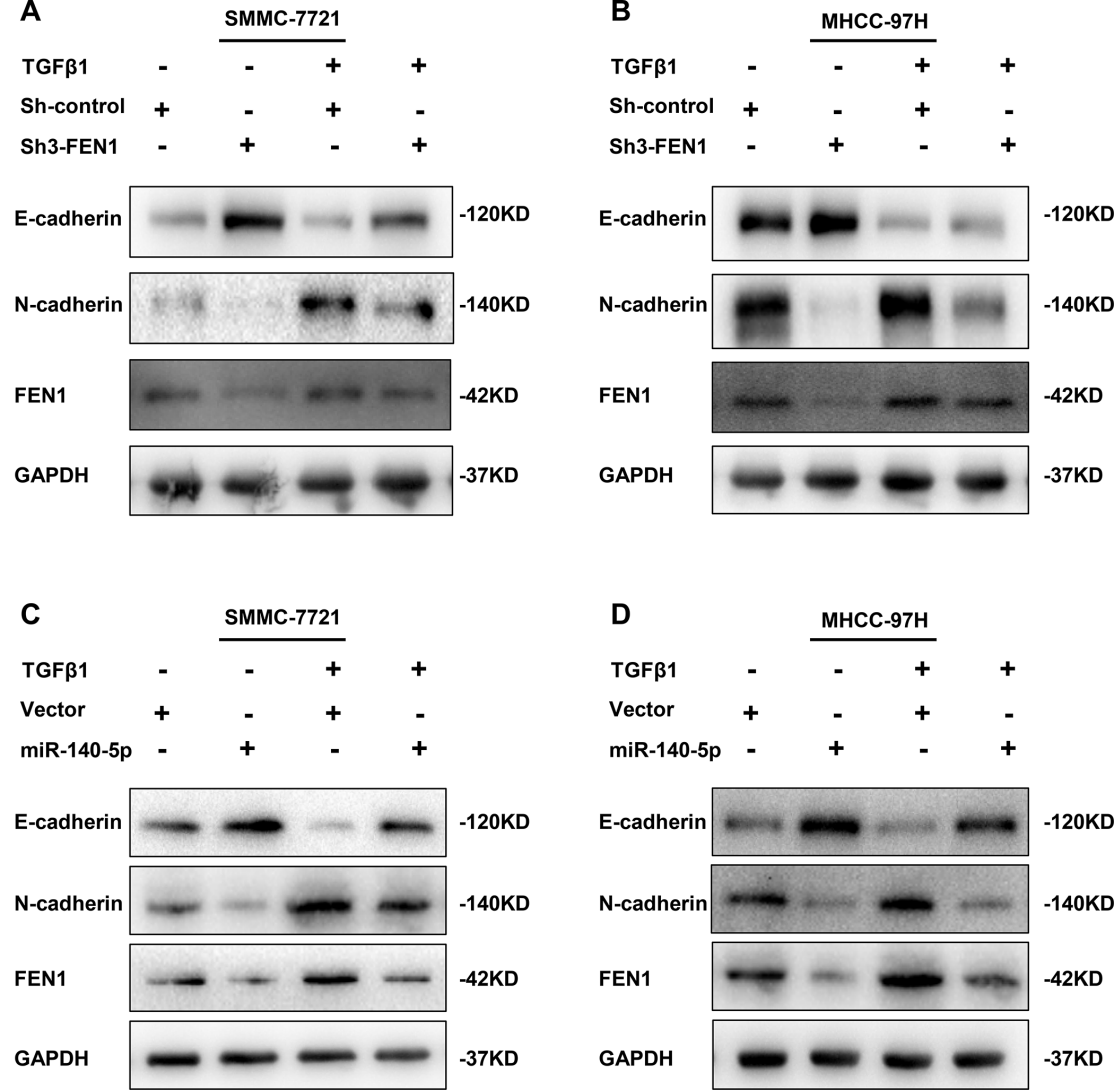
**TGF-β1 acts through miR-140-5p to up-regulate FEN1 in promoting EMT.** (**A** and **B**) Western blot analysis showing the effect of silenced FEN1 on EMT repression induced by TGF-β1. (**C** and **D**) Western blot analysis of the effect of miR-140-5p on the expression of established EMT markers and FEN1 in HCC cells after treatment with TGF-β1.

We confirmed that FEN1 was upregulated during TGF-β1-stimulated EMT in HCC cells. To further clarify the role of FEN1 in TGF-β1-induced EMT, we treated FEN1 silenced MHCC-97H and SMMC-7721 cells with TGF-β1 for 48 h. The results showed that the FEN1 knockdown reversed the TGF-β1-induced EMT phenotype including E-cadherin and N-cadherin expression (Figure 9A–9B). Taken together, these results suggest that FEN1 positively regulates TGF-β1- induced EMT in HCC cells.

### TGF-β1 acts through miR-140-5p to up-regulate FEN1 and promote EMT

**Figure 10:**
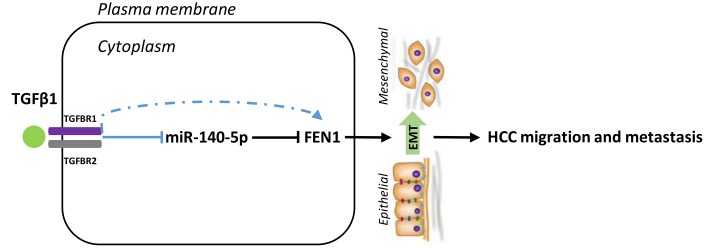
**Model for the TGFβ1-miR-140-5p axis in which up-regulation of FEN1 promotes HCC cells invasion and migration via accelerating the EMT process.** EMT, epithelial-mesenchymal transition.

miR-140-5p was downregulated in TGF-β1-treated HCC cells and its restoration reduced FEN1 expression. We hypothesized that miR-140-5p may oppose TGF-β1-induced EMT by decreasing FEN1 levels. Consistent with this hypothesis, the overexpression of miR-140-5p attenuated TGF-β1-mediated FEN1 upregulation (Figure 9C–9D). In addition, miR-140-5p could reverse TGF-β1-induced EMT (Figure 9C–9D). These data suggest that TGF-β1 induces FEN1 expression by inhibiting miR-140-5p, which promotes EMT processes (Figure 10).

## DISCUSSION

FEN1 maintains genomic stability through a variety of molecular mechanisms. Emerging evidence has shown that FEN1 dysfunction leads to genomic instability and increased cancer susceptibility [27, 28]. Reckamp and coworkers [29] reported a FEN1 E359K mutation that is oncogenic. In addition, FEN1 is a known biomarker for various cancers [17–21] but its expression in HCC tissue is unclear. We utilized bioinformatics analysis to investigate the expression of FEN1 in HCC. Our results showed that FEN1 was up-regulated in HCC and negatively correlated with HCC prognosis, indicating it as a potential prognostic marker for HCC. These results were validated in 34 paired liver cancer tissue samples. These findings are in agreement with studies showing that high FEN1 levels significantly correlate with cancer metastasis and tumor size [19, 20], but the underlying mechanisms by which FEN1 influences liver cancer metastasis remain unclear. In this study, FEN1 expression was significantly associated with metastatic status in multivariate analysis. Subsequently, we observed high FEN1 expression in metastatic HCC and six HCC cell lines compared to the normal human liver cell lines (HL7702). Of note, FEN1 was highly expressed in HCCLM3 which is a highly aggressive cell line, compared to MHCC97-H which demonstrates relatively low invasiveness. These results indicated that FEN1 plays a role in HCC metastasis.

Metastasis is a multistep process during which tumor cells leave the primary tumor, disseminate to distant sites, and form secondary tumors [2]. EMT plays pivotal roles in these steps to promote metastasis. During EMT, epithelial cells lose their polarized characteristics and acquire an invasive mesenchymal phenotype. As the EMT process progresses, the epithelial marker E-cadherin is down-regulated, but mesenchymal markers such as vimentin and N-cadherin are up-regulated. In this study, we demonstrated that FEN1 silencing inhibited HCC cell migration, invasion and EMT *in vitro*, and inhibited tumor growth and lung metastasis *in vivo*. FEN1 overexpression had the opposite effect, suggesting that FEN1 promotes EMT in HCC. This is in agreement with previous studies showing that FEN1 promotes cancer development and is required for S-phase entry in trophoblasts [30–32]. Mutations in FEN1 that disrupt its interaction with PCNA also induce aneuploidy-associated cancer [27]. Our results were consistent with the central role of FEN1 in cellular proliferation. The regulatory network of EMT is complex, and involves Wnt, RTKs, NF-kB, and TGF-β signaling. The mechanisms underlying FEN1 expression during EMT remained elusive.

MicroRNAs are small non coding RNAs of ~ 22–25 nucleotides in length, which can inhibit the translation and stability of the targeted mRNA, regulating genes involved in diverse cellular processes including apoptosis, migration and EMT. Previous studies confirmed that several miRNAs are involved in EMT processes including the miR-200 family [33]. Previous findings have also shown that miR-140-5p plays an important role in cell growth, invasion and metastasis of various cancer types, suggesting miR-140-5p acts as a tumor suppressor [34, 35]. Previous studies have shown that miR-140-5p is down-regulated in HCC and suppresses tumor growth and metastasis by targeting transforming growth factor β receptor 1 [36–38]. TGF-β signaling regulates embryonic development, cell proliferation, differentiation, and apoptosis [39]. As a key driver of EMT, TGF-β plays an important role in tumorigenesis and cancer metastasis, and induces EMT by activating transcription factors of the Snail, Slug, Twist and ZEB1 families [40]. Recent studies showed that miR-140-5p functions as a key suppressor of CRC progression and metastasis through inhibiting EMT processes through Smad3 [41]. However, the role of miR-140-5p in HCC cells during EMT has not been elucidated. Using the TargetScan database, we found that FEN1 is a direct target of miR-140-5p, which suppressed FEN1 expression in HCC cells which were restored with a miR-140-5p inhibitor. Moreover, a negative correlation was observed between FEN1 and miR-140-5p in HCC tissues. Consistent with previous studies, we provide evidence that miR-140-5p is down-regulated in HCC tissue which correlates with tumor metastasis and poor survival in HCC patients. Furthermore, we found that miR-140-5p attenuated HCC cell migration and invasion, and reversed the EMT processes induced by FEN1 overexpression. These data indicate that miR-140-5p suppresses HCC metastasis and EMT phenotypes by targeting FEN1, implicating TGF signaling in FEN1 mediated EMT.

To elucidate the underlying mechanism(s) by which FEN1 is involved in EMT, we explored the effects of TGF-β1 on the expression of FEN1 and miR-140-5p. Our results showed that TGF-β1 inhibited miR-140-5p expression and promoted FEN1 expression during EMT. A rescue study demonstrated that FEN1 silencing reversed the EMT process induced by TGF-β1. We also demonstrated that the downregulation of miR-140-5p was required for TGF-β-induced EMT, and that FEN1 was a direct target of miR-140-5p. These results indicated that miR-140-5p suppresses HCC metastasis and EMT phenotypes by targeting FEN1.

Given our findings, we reasoned that FEN1 has potential diagnostic and therapeutic benefits for HCC patients. Cisplatin is a potent anti-tumor agent. Recent studies showed that FEN1 depletion enhanced the cytotoxic effects of cisplatin in the lung [42], breast [43] and gastric cancer cells [44]. In addition, highly sensitive and specific nanoprobes were designed to regulate FEN1 activity in human cancer cells and acted as anticancer drugs [45].

In summary, we show that FEN1 is up-regulated in HCC tissue and human hepatoma cell lines compared to para-cancer normal liver tissues and normal hepatocyte cell lines. FEN1 promoted HCC cell migration and invasion through inhibiting EMT *in vitro*, and accelerated xenograft tumor growth and lung metastasis *in vivo*. Furthermore, our data suggest that overexpression of miR-140-5p in HCC cells inhibits EMT by down-regulating FEN1. TGF-β1 promoted an EMT phenotype, at least in part, by up-regulating FEN1 and down-regulating miR-140-5p. Based on these findings, we propose a model that highlights the role of FEN1 in regulating TGF-β signaling during liver cancer metastasis (Figure 10). Knowledge of the TGF-β1-miR-140-5p-FEN1 axis enhances our understanding of the TGF-β network and highlights FEN1 as a therapeutic target to dampen TGF-β signaling during liver cancer progression. The molecular mechanisms linking FEN1 and EMT are thus worthy of further investigation.

## MATERIALS AND METHODS

### TCGA data analysis

FEN1 expression in HCC was compared in the ONCOMINE database (https://www.oncomine.org). FEN1 expression and its relationship to clinical outcomes in HCC were analyzed using the GEPIA database (http://gepia.cancer-pku.cn/).

### HCC specimens

A total of 34 paired HCC and para-carcinoma tissue samples were collected from the department of hepatobiliary surgery in the Second Affiliated Hospital of Chongqing Medical University. Informed consent was provided by all patients. The study was approved by the Hospital Ethics Committee.

### Cell lines and culture

HL7702 and human the HCC cell lines SMMC-7721, HepG2, Huh-7, SK-HEP-1, MHCC97-H and HCCLM3 were obtained from the Department of Infectious Diseases, Institute for Viral Hepatitis, the Key Laboratory of Molecular Biology for Infectious Diseases, Chongqing Medical University. All cells were cultured in DMEM (Gibco, USA) supplemented with 10% fetal bovine serum (FBS; Corning, USA) in an incubator at 37°C with 5% CO_2_. The EMT phenotype was induced by treatment with 10 ng /ml recombinant human TGF-β1 (PeproTech) for 48 h.

### Establishment of stable cell lines

Viral vectors were produced by Obio Technology Corp., Ltd (Shanghai, China) including pLenti-EF1a-luc-F2A-Puro-CMV-FEN1-3FLAG and pLKD-CMV-G&PR-U6-shRNA-FEN1. We also purchased a lentiviral vector containing primary transcripts of miR-140-5p (Applied Biological Materials, Canada). Small interfering RNA (shRNA) sequences targeting human FEN1 were designed and are listed in Supplementary Table 1. Primer sequences for FEN1 overexpression vectors are listed in Supplementary Table 2. Transfections were performed according to the manufacturer's protocols. Briefly, cells grown to ~40% confluency were infected at a multiplicity of infection (MOI) of 40 using 5 μg/ml polybrene to aid viral attachment. Cells were selected with 2 μg/mL puromycin for a minimum of 4 weeks to select stably transfected lines.

### MiR-140-5p knockdown

To silence miR-140-5p expression, ~2 × 10^5^ cells were transfected with 100 nM miR-140-5p inhibitor using riboFECT™ CP Reagent (RIBOBIO, Guangzhou, China) in six-well plates, according to the manufacturer’s instructions. Transfected cells were harvested at 72 h post-transfection.

### Real-time fluorescence quantitative PCR (RT-qPCR)

Total RNA was extracted using the phenol chloroform method. Complementary cDNA synthesis was performed using an RT Reagent Kit with gDNA Eraser (Takara, Japan) according to the manufacturer’s protocol. Quantitative RT-qPCR was performed using SYBR Premix ExTaq (Takara, Japan) and the CFX96 Real-Time PCR Detection System (Bio-Rad, USA). Glyceraldehyde 3-phosphate dehydrogenase (GAPDH) and U6 were used as internal controls. Primers are listed in Supplementary Table 3. Results were analyzed using the 2^-ΔΔCt^ method.

### Western blot analysis (WB)

Protein extraction and quantitation were performed using kits obtained from KeyGEN BioTECH, China. Supernatants were resolved on 10% SDS/PAGE and transferred to 0.45 μm PVDF membranes (Millipore, Billerica, MA). Membranes were blocked with 5% Bovine Serum Albumin (GENVIEW, USA) for 1 h at room temperature and probed with the indicated primary antibodies at 4°C overnight. Membranes were then washed and labeled with secondary antibodies for 2 h at room temperature. Blots were visualized using the BIO-RAD imaging system (BIO-RAD, Hercules, CA, USA). The following antibodies were used: FEN1 (A1175, ABclonal, China), E-cadherin (ab40772, Abcam, Cambridge, UK), N-cadherin (ab76057, Abcam, Cambridge, UK), Vimentin (ab92547, Abcam, Cambridge, UK), MMP2 (ab92536, Abcam, Cambridge, UK), MMP9 (ab76003, Abcam, Cambridge, UK), Anti-rabbit IgG, HRP-linked Antibodies (#7074, Cell Signaling Technology, Danvers, MA, USA). GAPDH was used as a loading control (#5174, Cell Signaling Technology, Danvers, MA, USA).

### Dual luciferase reporter assay

The bioinformatics analysis algorithm TargetScan (http://www.targetscan.org/vert_72/) was used to predict miR-140-5p binding sites of the FEN1 fragment. The miR-140-5p binding site in the 3′-untranslated region (3′-UTR) of FEN was cloned into the pMIRREPORT vector (Promega) to produced pMIR-FEN1-Wt (FEN1-Wt). The putative miR-140-5p binding site in FEN1 was mutated and the sequence was named FEN1-Mut. FEN1-Wt and Mut were co-transfected with miR-140-5p mimics into HEK-293 cells in 96-well plates using Lipofectamine 3000 (Invitrogen). After 48 h, Renilla luciferase activity was detected using a Dual-Luciferase Reporter Assay System (Promega). Firefly luciferase was normalized to the Renilla signal.

### Wound healing assays

Stably transfected cells were seeded into 6-well plates at a density of 2×10^5^/ml and cultured until confluent. A wound gap was scratched using a 10 μL pipette tip and cell migration was assessed following incubation in serum free media for 48 hours. Images were acquired using an inverted phase microscope (Nikon, Japan). Migration distances were calculated using Image-Pro Plus 6.0 software (IPP, Media Cybernetics, Rockville, MD, USA) and the following formula: Distance = D_0h_-D_48h_.

### Transwell assays

For migration assays, approximately 2×10^4^ SMMC-7721 or 1×10^5^ MHCC-97H cells suspended in 200 μL serum-free medium were seeded into the top chamber with 8.0-μm pores (BD Bioscience, USA). For invasion assays, cells seeded in the top chamber were coated with matrigel (Corning, USA) in 24-well plates. Matrigel was diluted in serum-free medium at a ratio of 1 to 8. DMEM supplemented with 10% FBS (700 μl) was added to the bottom chamber. Cells at the undersurface of the chamber were imaged and counted.

### Immunohistochemistry (IHC)

Paraffin sections were baked at 56 °C for 2 h for dewaxing, boiled in citrate buffer for antigen retrieval, and blocked using 3% hydrogen peroxide. Sections were probed overnight at 4°C with anti-FEN1 (A1175, Abclonal, China), anti-E-cadherin (ab40772, Abcam, Cambridge, UK), anti-N-cadherin (ab76057, Abcam, Cambridge, UK), and anti-Vimentin (ab92547, Abcam, Cambridge, UK) antibodies, followed by incubation with HRP-conjugated secondary antibodies for 1 h at 37 °C. Sections were stained using DAB coloration kits and counterstained with hematoxylin for 10 s. Samples were dehydrated using a graded series of ethanol. Image-Pro Plus software was used to measure IHC staining intensity. IHC scores were calculated by multiplying the percentage score and intensity score of stained cells. The percentage of positively stained cells was scored on a scale from 0 to 100. Staining intensity (0 = negative staining; 1 = weak; 2 = moderate; 3 = strong) was scored on a scale from 0 to 3. Thus, the IHC score ranged from 0 to 300. The mean IHC score was defined as the cut-off value for low and high expression.

### Animal studies

All experiments with BALB/c nude mice were performed in accordance with the National Animal Experimentation guidelines and approved by the Ethics Committee. Four-week-old nude mice were purchased from BEIJING HUAFUKANG BIOSCIENCE CO.INC (Beijing, China) and maintained in a specific-pathogen-free (SPF) barrier environment. Cell lines (approximately 1 × 10^6^ in 100 μL saline) were subcutaneously injected into the armpit, along with the corresponding control cells (n = 5 mice per cell line). The largest (A) and smallest (B) diameter of the tumor were measured using a vernier caliper every 3 days. Tumor volumes (V) were calculated according to the formula: V= ½ × AB^2^ (mm^3^). Mice were executed after 4 weeks and tumor weights were measured. The lung metastasis tumor model was established by injecting cells (approximately 2 × 10^5^ in 200 μL saline) into the tail vein of nude mice. Weights of the nude mice were monitored every 3 days

### Statistical analysis

Statistical analysis was performed using SPSS 19.0 software (SPSS Inc, USA) and Graph Pad Prism 8.0 (Graph Pad Software, USA). Data were acquired from at least three independent assays and are shown as the mean ± standard deviation (SD). Chi-Square tests were used to analyze the correlation between FEN1 and clinicopathological features. The correlation between miR-140 and FEN1 expression was evaluated using Spearman’s correlation analysis. Multiple datasets were compared using a one-way analysis of variance (ANOVA). Paired tumor and normal tissue data were compared using a paired Student’s t test (two-sided). A P < 0.05 was considered statistically significant (*P < 0.05; **P < 0.01).

## Supplementary Material

Supplementary Figures

Supplementary Tables
