# Supplementary material for: TGFβ1- miR-140-5p axis mediated up-regulation of Flap Endonuclease 1 promotes epithelial-mesenchymal transition in hepatocellular carcinoma
Source: Aging (Albany NY). 2019 Aug 10;11(15):5593–612. doi: 10.18632/aging.102140 (PMC6710057; doi:10.18632/aging.102140)
Supplement: Supplementary Figures [file aging-11-102140-s001.pdf]

SUPPLEMENTARY FIGURE

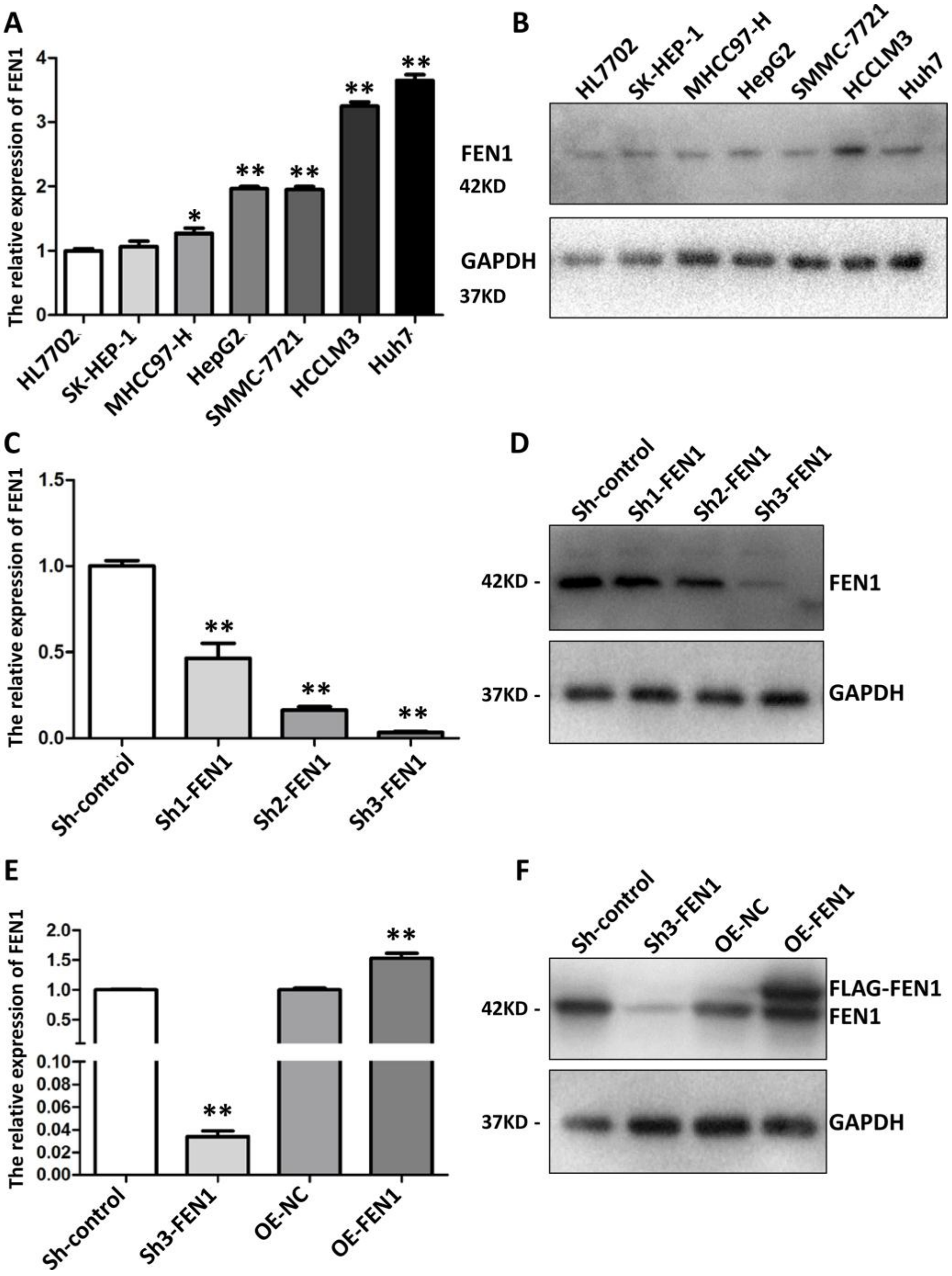

Supplementary Figure 1. FEN1 is significantly upregulated in hepatoma cell lines and stable FEN1 knockdown and overexpression lines were established. (**A**) RT-qPCR analysis of FEN1 mRNA expression in normal human liver HL7702 cells and six hepatoma cell lines (SK-HEP-1, MHCC97-H, HepG2, SMMC-7721, HCCLM3 and Huh7). (**B**) Representative WB analysis of FEN1 protein levels in HL7702 and the six hepatoma cell lines. (**C**) RT-qPCR analysis of FEN1 mRNA expression after lentiviral expression of the different ShRNA-FEN1 constructs. (**D**) Representative WB analysis of FEN1 expression after lentiviral expression of ShRNA-FEN1. RT-qPCR (**E**) and WB (**F**) analysis were used to identify successfully generated cell lines. All data represent mean ± SD of three independent experiments; compared to HL7702 cells or the corresponding control group, *$P < 0.05$ and **$P < 0.01$.
