# Supplementary material for: TGFβ1- miR-140-5p axis mediated up-regulation of Flap Endonuclease 1 promotes epithelial-mesenchymal transition in hepatocellular carcinoma
Source: Aging (Albany NY). 2019 Aug 10;11(15):5593–612. doi: 10.18632/aging.102140 (PMC6710057; doi:10.18632/aging.102140)
Supplement: Supplementary Tables [file aging-11-102140-s002.pdf]

SUPPLEMENTARY TABLES

Supplementary Table 1. The sequence of small interfering RNA (shRNA) targeting human FEN1.

shRNA	Target sequence
Sh-control	TTCTCCGAACGTGTCACGT
Sh1-FEN1	GCCCGTGTATGTCTTTGAT
Sh2-FEN1	CCTCATGGGCATCCCTTAT
Sh3-FEN1	GCTCACTCTCTTCAGCTAA

Supplementary Table 2. Primer sequences for FEN1 overexpressing vector.

Gene	Primer sequences
FEN1	CMV-F CGCAAATGGGCGGTAGGCGTG
	WPRE-R CATAGCGTAAAAGGAGCAACA

Supplementary Table 3. Primer sequences for quantitative RT-PCR.

Gene	Primer sequences
FEN1	Forward (5′-3′) : CTGTGGACCTCATCCAGAAGCA
	Reverse (5′-3′): CCAGCACCTCAGGTTCCAAGA
GAPDH	Forward (5′-3′) : GGTGGTCTCCTCTGACTTCAACA
	Reverse (5′-3′): GTTGCTGTAGCCAAATTCGTTGT
hsa-miR-140-5p	Forward (5′-3′) : CGCGCGCAGTGGTTTTACCCTA
	Reverse (5′-3′): ATCCAGTGCAGGGTCCGAGG
	RT Primer: GTCGTATCCAGTGCAGGGTCCGAGGTATTCGCACTGGATACGACCTACCA
U6	Forward (5′-3′) : AGAGAAGATTAGCATGGCCCCTG
	RT Primer: GTCGTATCCAGTGCAGGGTCCGAGGTATTCGCACTGGATACGACAAAATA

Supplementary Table 4. FEN1 expression and clinicopathological features in 34 paired HCC patients.

Clinicopathological features	Cases (n=34)	FEN1 expression		*P* value
		High (%)	Low (%)	
**All case (n=34)**	34	25	9	
**Gender**				1.000
Male	29	21(61.8%)	8(23.5%)	
Female	5	4(11.8%)	1(2.9%)	
**Age(y)**				0.403
<45	8	7(20.6%)	1(2.9%)	
≥45	26	18(52.9%)	8(23.5%)	
**Tumor size**				0.047
<5cm	19	11(32.4%)	8(23.5%)	
≥5cm	15	14(41.2%)	1(2.9%)	
**Tumor stage**				1.000
I ~ II	23	17(50.0%)	6(17.6%)	
III ~ IV	11	8(23.5%)	3(8.8%)	
**Differentiation**				1.000

Well/moderate	27	20(58.8%)	7(20.6%)	
Poor	7	5(14.7%)	2(5.9%)	
**Metastasis**				0.013
With	12	12(35.3%)	0(0.0%)	
Without	22	13(38.2%)	9(26.5%)	
**HBsAg**				1.000
Positive	30	22(64.7%)	8(23.5%)	
Negative	4	3(8.8%)	1(2.9%)	
**Liver cirrhosis**				0.687
With	12	8(23.5%)	4(11.8%)	
Without	22	17(50.0%)	5(14.7%)	

Note: *P* value is calculated with χ^2 test.
